# Subjective Can Be Scary—But Worth It: Personal Reflections on How Qualitative Methods Can Advance Applied Behavior Analysis

**DOI:** 10.1007/s40617-025-01120-6

**Published:** 2025-11-23

**Authors:** Suzy Mejía-Buenaño

**Affiliations:** Care and Outcomes Research Centre, University of Kent, Canterbury, UK

**Keywords:** Qualitative research, Applied behavior analysis, Narrative inquiry, Behavior analyst

## Abstract

Applied behavior analysis is a quantitative field. We calculate frequency of responses per minute, percentage of incorrect and correct responses, percentage of agreement across raters on a regular basis. The safety in numbers can be comfortable—they are clear and objective. However, numbers do not provide the whole picture of a person’s experience. Qualitative approaches provide valuable insights into the lived experience of people. Yet, undertaking qualitative approaches can be scary for those of us in a quantitative field. The subjective data and findings can be extremely challenging to navigate. There is also the matter of feeling like an imposter or a fraud. In this personal narrative inquiry, I tell my story of embracing qualitative approaches as a behavior analyst, the challenges and the surprising discoveries of the depths this data could help us reach. The relevance of qualitative approaches lies in understanding how various qualitative methods and approaches can enhance our understanding of lived experience. Some points about qualitative research are drawn out for context, and my personal experience is explored to show the journey, joys, and challenges of discovering and embracing qualitative research as a behavior analyst.

## Introduction

Qualitative research came into my career unexpectedly. I was always a quantitative thinker. Hard, cold numbers and facts. That was what I envisioned for my research career, and the heavy emphasis on data collection and analysis (based on numbers, of course) was part of what drew me to applied behavior analysis (ABA). However, my PhD was centered on research questions that needed to be answered using qualitative methods. I was taking an inductive approach to my research and one of my research questions obligated an in-depth exploration of personal family experiences, and qualitative methods were the most appropriate for this. So, I had to pivot. I had to learn about qualitative methodologies and embrace the discomfort I felt with subjective data. And on that journey, I learned skills that to my surprise made me a better clinician, researcher, and advocate for people with disabilities and their families. At the time, I had not anticipated that learning qualitative research skills would improve my work as a clinician because I did not know how qualitative research skills fit within ABA. While this has been life-changing for my practice and my research, I was left feeling like an imposter in our quantitative-heavy field. In this paper, I adopt a personal narrative inquiry (Kim, 2016; Langellier, 1989) to share the story of my own qualitative research experiences while incorporating an analysis of how my experiences intersect with the broader context of our behavior analytic field (e.g., Kim, 2016; Richardson & St. Pierre, 2005).

Personal narrative is an approach whereby a researcher creates a narrative about their own personal experience (Yuan & Hickman, 2016) to provide an insight to personal experiences with scholarly interpretation (Kim, 2016). Narrative inquiry is based on the premise that telling stories or narratives about the things we experience is an innate part of our upbringing and experience as people, and that personal narrative is a communication method that transcends all disciplinary boundaries (Langellier, 1989). This approach falls under the umbrella of autoethnographic approaches in which personal experiences are used to explore and/or critique social or cultural experiences (Jones et al., 2013). This paper follows my journey through five phases: I describe the start of my journey with qualitative methods and the challenges it brought up for me, the experience of diving into qualitative methods, seeing how these fit within our work in the field of ABA, and ways in which adopting qualitative methods can help move our field forward. I conclude with some final personal reflections. I hope that by inviting you into my world and my experiences, you find a way to reflect on your own practice, and sparks for conversations with students, colleagues, and researchers about the place of qualitative methods in our field.

## Phase One: The Start of My Qualitative Journey in Research

My journey with qualitative research began when I started my PhD. As mentioned, I had always been more of a quantitative thinker, drawing a sense of comfort in numbers and facts. But my PhD was in behavioral feeding difficulties—an area that has much research evidence demonstrating the impact and utility of behavioral interventions (e.g., Ahearn, 2003; Alaimo et al., 2018; Berth et al., 2019), and, at the time, not such an extensive understanding of parent or family experiences (e.g., Adams et al., 2020; Estrem et al., 2018; Rogers et al., 2012). One of my research questions was about understanding the experiences of parents when they have a child in the family with a behavioral feeding difficulty. Given the inductive nature of my PhD research, this meant I needed to explore family experiences of behavioral feeding difficulties before I could move onto thinking about intervention development. It was evident that this would be central in my PhD journey since there was not at the time much research from which to develop an understanding of the family experience of mealtime challenges. Thoughts similar to those below circled in my mind continually during this period:

*This is going against my training as a behavior analyst! My goal is to develop interventions that have contextual fit with families’ lives and to help them have long-term change in mealtimes, how can I do that without really understanding what their mealtime experiences are like?*

I explored various research methodologies I could use to design a study that would help me develop this understanding. I could have taken various approaches: interviews, a survey, mealtime observations, to list a few. However, interviewing parents stood out as a valuable way to learn about families’ experiences of their children’s behavioral feeding difficulties. Through interviewing, I could take a more open approach, letting families tell their stories while asking probing questions (i.e., a semi-structured approach). Interviews with a semi-structured approach are a powerful research method which allow information to be gathered from stakeholders in a flexible way, enabling potentially sensitive topics to be explored in depth (DeJonckheere & Vaughn, 2019).

It seemed to me that this was the appropriate research method to gather the data that would help me to answer the research question for the first study in my PhD. But I didn’t *want* to interview families. I was scared about the uncertainty of how things would go in a research interview. I had worked as a research assistant before, and ran experiments in a lab, using direct and comprehensive task analyses to guide my work. But this didn’t translate in the same way in qualitative interviews. The idea of doing this as a data collection method felt foreign. Like I was turning my back on my objective, data-focused training. I felt like a traitor to ABA and like an imposter as a behavior analytic academic. Being a behavior analyst and conducting subjective research! I dreaded to think what behavior analysts completing PhDs would think about my “flouncy” qualitative project and its subjective findings. This narrative sowed seeds of self-doubt:

*I don’t see many behavior analysts conducting qualitative research, are my instincts about the value of this type of data wrong?*

*I am teaching the next generation of behavior analysts in the UK! How can I be taken seriously when I’m conducting subjective research and teaching about objective data collection…*

As my PhD evolved, it dawned on me that I would need not only one qualitative research project, but three. Realizing this exacerbated the thoughts I was having. During this time I felt like a fraud. All my behavior analytic training, and *three* studies worth of subjective data and analysis. I distinctly recall having conversations with other behavior analysts about my qualitative research and feeling like I needed to justify why I was conducting so much qualitative research in the context of a behavior analytic PhD. In those moments, I felt like a small mouse hunted by a hungry cat. There were of course additional intersectional layers which added to this feeling—being a young woman in academia and being the only ethnic minority in a predominantly white environment. However, these were intersectional layers which I had encountered before in other contexts; having this feeling in the context of the field of ABA was new. I had always viewed the ABA community as a group of like-minded individuals, yet I felt not so like-minded to the field anymore.

Feeling like an imposter in academia is not uncommon (Bothello & Roulet, 2019; Wilkinson, 2020). In fact, the very nature of feeling like an imposter is something that has been well-studied (e.g., Chapman, 2017; Freeman & Peisah, 2021; Rivera et al., 2021, etc.). Intersectionality is another well-studied theoretical framework that highlights the ways in which multiple identities (e.g., race/ethnicity, gender, etc.) come together to influence the experiences one has as they walk through the world (Crenshaw, 1989). I understood that the feelings I had with regard to my experiences and feelings of self-doubt as a qualitative researcher in ABA were intertwined with my other “identities”: young, female, and an ethnic minority. And while I found comfort in knowing that I was not alone in feeling like an imposter, feeling this way in the field of ABA was new and uncomfortable.

## Phase Two: Diving into the Depths and Unexpected Discoveries

Despite my feelings of discomfort, I pushed on with my qualitative research projects. At first it felt foreign. But once I had completed some interviews and listened to parents openly about their experiences of their child’s feeding difficulty, I realized just how valuable these interviews were. I was hearing stories that were heartbreaking, inspiring, and importantly, gave me rich insight into what life was like for my participants. I immediately realized how powerful these methods could be to enhance our understanding of a family’s situation.

I was particularly impacted by one parent’s story, which has remained with me from that first interview and significantly influenced my ongoing work in the area of behavioral feeding difficulties. I first met Charles (pseudonym) via a Teams call on a gloomy weekend evening, during the depths of a COVID-19 lockdown in November of 2020. Charles’ fifteen year old son, Adam, (pseudonym) had a severe intellectual disability and a severe feeding difficulty which had been unsuccessfully addressed by the relevant professionals in the UK. Charles had fought relentlessly to get Adam appropriate support, but Charles described being met with dismissal by medical professionals who he reported did not dig deeper to identify why Adam was refusing food. Charles shared with me that Adam only drank a supplemental milk drink from a baby bottle. There had been repeated failed attempts to move Adam onto a drink receptacle that was more age appropriate, like a sports bottle, and to encourage food consumption. Charles and his wife were at their wits’ ends, and desperate for a solution. Adam was already in residential care and would be transitioning to adult services in a few years. Charles and his wife were seriously concerned about whether Adam would be able to “survive” in an adult residential service for people with severe intellectual disabilities.

*The main focus has always been just keep him alive, you know, because the next step is to cut a hole in him and put a stent into his stomach, which is what they wanted to do… [they] told us that’s the eventual destiny for him. And he wouldn’t have survived that…he would have pulled it out and, you know, ripped his guts out. We wouldn’t be able to stop him. So, yeah, I mean, you know, better that he drinks even a baby bottle badly, than have a stent put in his stomach.*^1^

This fear was valid, given repeated scandals in the UK about neglect and abuse of vulnerable adults with intellectual disabilities (BBC News, 2012; BBC News, 2019; ITV, 2025). I left this interview feeling helpless and outraged for Charles and Adam. I could not do anything to help them in that moment other than to signpost them to support resources, as was outlined in my ethical approval from the University of Kent. I also felt indignation at the thought that so many professionals had been unable to provide any support which improved Adam’s feeding. I was not particularly surprised as, at the time, behavior analytic services in the UK were present but not prominent and significantly lacking in the context of feeding difficulties. But I was horrified to realize the full extent of the impact of having insufficient support in an area as important as feeding difficulties. While this is Charles and Adam’s story, the sentiment was echoed by 20 other families I interviewed for this study.

Their story made a significant impact on me as a researcher. I had, perhaps naively, not realized just how open participants in research interviews could be. I was entirely caught off guard by Charles’ honesty, particularly his description of the grave implications of the situation for Adam. I was aware going into the interviews that I would be learning about people’s experiences, but the reality of hearing the personal stories themselves was *not* something the textbooks prepared me for. The gravity of Charles and Adam’s story entirely shifted my perception of the insights that research interviews could bring. I felt humbled and privileged that families shared their deeply personal stories with me. This was an important turning point–I was no longer scared of the subjectivity of qualitative research. I had seen its value.

## Phase Three: Fitting It in with Our Current Work

To my surprise, as my PhD progressed, I started seeing how aspects of what I was learning about qualitative methods related to ABA. As with my broader PhD, in the study described above I was taking a “bottom up” or inductive approach. I was following the data and drawing conclusions directly from participants’ data. I realized that this was not so unfamiliar to the inductive approach we often take in ABA. Qualitative research often takes an inductive approach, where meanings and themes are derived directly from the data rather than from pre-identified theoretical frameworks (Thomas, 2003) and inductive methods are commonly used in qualitative studies (e.g., Fernández-Alcántara et al., 2017; Inan Budak et al., 2018; Knox et al., 2000). There were some key design features of inductive studies which I immediately realized had relevance to my practice as a behavior analyst. For example, understanding the differences between semi-structured and structured interviews could support clinicians in obtaining richer assessment data. In a structured interview, a standardized list of questions and follow-up probes is used, the questions are delivered verbatim to participants, they are asked in the same order, and participant responses may be rated in a systematic way (Mueller & Segal, 2015). Structured interviews are considered to yield more easily comparable participant responses (Brinkmann, 2014). Semi-structured interviews share some aspects of structured interviews, but deviate from these in key ways. For example, pre-determined questions may be posed verbatim to participants, but there is flexibility in the ability to follow-up on participants’ responses to these main questions (Mueller & Segal, 2015). With structured interviews, there is no room to follow the participant with what they share, whereas with semi-structured interviews, the interview questions are used more like a guide, and this allows an interviewer to follow a participant’s lead. Semi-structured interviews allow the interviewer flexibility and allow for a more inductive approach to be taken to data collection in the assessment process. When using the Functional Assessment Interview (O’Neill et al., 1997), for example, the use of the interview schedule as a semi-structured one rather than a structured one could yield richer data. As a field, we are established in taking an inductive approach (Locey, 2020), and this can be extended to data collection in the assessment stage of our clinical practice. As I gained experience with interviewing, I realized there was more relevance in qualitative methods to ABA than I had ever considered.

I also developed interpersonal skills in my qualitative interviewing, which supported my development as a clinician. For example, in my experience, qualitative interviewing requires active listening (Lavee & Itzchakov, 2023; Louw et al., 2011), reflective statements, and the ability to comfortably sit in silence. Active listening is an important skill for behavior analysts, and while our technical training provides robust insight and knowledge about behavior change, it has been my experience that these skills are not often directly taught or obtained through observational learning on the job. The benefit, as I saw it, of conducting qualitative research in my PhD was that I received specific training on this skill, which I believe later supported my clinical work. Active listening is generally considered to be a critical component of communication rooted in empathy among those working in the helping professions (Tustonja et al., 2024). In fact, research has shown that active listening can foster a supportive conversation between allied health professionals and clients. For example, Cameron et al. (2018) used qualitative methods to explore how clinicians foster engagement in rehabilitation patients to set patient-centered goals. They found that clinicians described active and reflective listening as allowing patients to direct the conversation to the matters of most importance to them and to support with goal setting. Reflective statements are commonly used in counselling, and these include restatements, reflections and open-ended questions (Rautalinko, 2012). Reflective statements can also be used in qualitative research to clarify understanding and observe a participant’s experiences (e.g., “it sounds like that was a challenging time”). This also has relevance for ABA as the use of reflective statements in an assessment process, or during check-ins with families, may help to ensure both parties have a shared understanding of the situation or identify challenges families may not feel comfortable explicitly stating (e.g., “it sounds like the original plan is not going as hoped”, etc.). Finally, in my research interviews, I quickly learned that being comfortable with silence is a crucial interpersonal skill when interviewing as it allows participants time to gather their thoughts. I gathered that the same premise would be helpful in clinical work. With increased workloads and pressure, particularly in a climate in ABA where burnout can be high (Plantiveau et al., 2018), it can be easy to rush things. However, giving people the space to gather their thoughts and think about whether they want to add something may be a powerful tool in obtaining the full picture of their experience, perceptions, or preferences. The discussion around interpersonal communication skills is one of particular importance for behavior analysts. Recently, Bailey and Burch (2023) emphasized the importance of interpersonal communication skills, including active listening, in behavior analytic practice. We work at the interface between society and science, and while science is the backbone of our field, which we are subject to rigorous training for, the empathetic side of our work is not often specifically taught. Nevertheless, learning about qualitative methods and qualitative interviewing can support with developing these transferrable skills. Behavior analyst training programs may consider the benefit of encouraging students to complete dissertations or theses using additional methodologies to single-case design. This may encourage students to develop a broader range of skills, which may help equip them with the interpersonal communication skills required as behavior analysts.

Previous work has identified the relevance of qualitative methods for exploring social validity (Burney et al., 2023; Guinness et al., 2024). Other areas of our practice may also benefit. Assessing contextual fit is one example. Cheremshynski et al. (2013) used qualitative methods to explore the experiences of parents and interventionists of implementing a culturally appropriate positive behavior support plan. The findings demonstrated the power of achieving contextual fit and provided insight into the mother’s experience and perspective on their family life. They presented these findings from the perspective of Emi, the mother of the autistic participant receiving behavioral support, and from the perspective of the interventionist. Emi expressed hopes for her children and reflections on life stressors. The findings provided Emi’s perspectives on the behavior support plan, and this showed that the plan developed was a good fit with the cultural beliefs and values of the family. The interventionist’s qualitative findings highlighted the importance of building and maintaining rapport for a culturally responsive behavior support process; obtaining guidance from a cultural interpreter to acquire a better understanding of the family; and recognizing and accommodating a cross-cultural belief system. This paper provides an excellent example of the value of qualitative data and the insights into contextual fit this can provide.

Qualitative methods can yield information about the rich lived experience of participants. This is another way in which qualitative methods can provide useful insight to our practice as behavior analysts. This is particularly important for those working in practice with individuals with intellectual disabilities or autistic individuals and their caregivers. In our work, we come into people’s lives at a time of need with the aim of providing support. Understanding the lived experience of those we work with can help ensure our work is appropriate and meaningful to those we support. For example, (Griffith, et al., 2013) conducted a qualitative systematic review of research in this area and found that caregivers reported experiencing chronic strain due to the demands of caregiving that they experienced. This insight is relevant for behavior analytic practitioners, as it provides understanding into the context of being a caregiver for someone with a disability, and the importance of developing interventions which will fit into their family lives, as opposed to creating additional strain. My experience with conducting qualitative research and analyzing qualitative data in relation to behavioral feeding difficulties has been similar. While the research literature provided substantial information regarding the intervention process, there was little information about *how* families experienced behavioral feeding difficulties. Clinicians working with feeding difficulties in practice have this knowledge by virtue of their experience. But for clinicians *not* specializing in feeding difficulties who encounter a client with a feeding difficulty in practice, the research literature, along with clinical supervision, are often used as a guiding point, yet the research literature does not appear to fully unpack this family experience. Additionally, exploring the lived experience of individuals with intellectual disabilities or autistic individuals themselves would yield rich and useful insights for behavior analysts.

Knowing the importance of understanding lived experience also allows us to reconsider the research that we carry out in the field of ABA. For example, there has been significant research looking at various applications of functional communication training (Ghaemmaghami et al., 2021). Areas of research on functional communication training have explored the use of behavioral skills training to train staff in implementing functional communication training (King et al., 2025) and procedural variations like discriminated functional communication (Kuhn et al., 2010), though this is not an exhaustive list. While functional communication training has been researched from various angles, there may also be value in researching the lived experience of caring for someone with communication challenges or being a person with communication challenges. As an example, understanding the experience of caregivers of children with communication challenges may provide insight about their personal challenges in navigating their child’s needs, and this could be very important when developing interventions to develop functional communication, as it can support a focus on contextual fit. There has been research exploring the lived experience of caring for someone with communication challenges or being a person with communication challenges (see Bradshaw et al., 2013, etc.); however, research from a behavior analytic perspective is likely to yield unique insights. This also links to initiatives in the field of intellectual disability to increase co-production in research (e.g., Griffin et al., 2023; Sutherland et al., 2024), which is another way that qualitative methods can further our research in ABA.

## Phase Four: Moving the Field Forward

While feeling like an imposter felt like my own personal isolated experience, I realized that it was unlikely that I was the only person in the field to have ever felt this way. I could see other behavior analysts working with different populations or applying their knowledge in entirely different fields and wondered if they had felt like imposters at times as well. For example, many behavior analysts work with autistic people or people with intellectual disabilities (e.g., Conine et al., 2025; Fallon et al., 2025; Walker et al., 2025, etc.), and it is possible that others working in areas outside of autism and intellectual disability might also feel like imposters in the field. For example, those working in organizational behavior management may feel alienated when much of the field focuses on autism or intellectual disability.

Interestingly, while change can be difficult, there have been recent examples of clinicians and researchers pushing the boundaries of ABA in relation to qualitative methods. For example, Burney et al. (2024) published an article discussing the ways in which qualitative methods can be applied in ABA research. They argue that qualitative approaches provide us with specific skills which enable us to center the voice and experiences of participants, as well as provide a contextual understanding of situations. Additionally, Burney et al. (2023) also argued that qualitative approaches may support our field in evidencing the outcomes from behavior change interventions, from the perspectives of stakeholders. As someone who has felt on the fringes of the field by virtue of my focus and developing expertise in qualitative methods, I have found it particularly encouraging to see recent narratives and focus on qualitative research and its place in our field.

I found hope in seeing other researchers in ABA embracing qualitative approaches and some increased discussion about this, but it did not shake the feeling that I was screaming into the abyss about the importance and relevance of qualitative approaches. In my experiences of teaching behavior analysis, when discussing relevant qualitative research and qualitative methods, I was not uncommonly met with blank stares from my behavior analysis students. There were few and far between moments where I saw nods of recognition and understanding in regard to the value of qualitative methods. Of course, the Behavior Analyst Certification Board guides the teaching on Verified Course Sequences, and there is little opportunity to expose students to research methods other than single-case design methods, so it is unsurprising that these methods would feel foreign to some. There are clearly some broader systemic challenges at play. While there may have been distinct reasons for why some students responded in this way, the feeling of screaming into the abyss about the importance and relevance of qualitative methods persisted. In encounters with other members of the field, some have embraced my insights about qualitative research and its applications within our field, while others have struggled to see the relevance. I fear that any rigidity toward maintaining data collection and analysis centered on hard facts and numerical data will stop the field from moving forward, as it pertains to the relevance of qualitative approaches. Data are the bread and butter of our field, and we all know the ways in which these can change lives. But data can also look different, and have just as much of an impact, when it is in the form of words, rather than tallies or percentages. Valuing subjective data does not make us worse behavior analysts. Subjective data can provide a direct insight into personal experience which can enhance our clinical work. Clearly, the distinction between the objectivity of numbers and the subjectivity of words as data was not as binary as it appears.

Carrying out qualitative research can also lead us to asking broader questions. We are an inductive field, and this should also extend to our research questions. Qualitative approaches allow us to pose open and exploratory research questions and to follow a “bottom up” approach. As discussed previously, the development of interpersonal communication skills may be able to be at least partially addressed by training and experience in conducting semi-structured interviews, including active listening, and making reflective statements. Teaching these transferrable skills is another way to change the field, the focus and scope of some of our research, and change our practice for the better.

## Phase Five: Some Personal Final Reflections

As a field that places value on operational definitions and technological procedures, the subjectivity and flexibility that a qualitative approach requires can be unfamiliar and scary. Applied behavior analysis is a quantitative field, and it is likely to be uncomfortable to shift to learning and accepting a subjective research method (if conducting research) or data collection and analysis method (if applying this in practice). However, in my experience of conducting and teaching qualitative methods, these methods have the potential to increase the value, impact, and relevance of our behavior analytic work and research. They will push us to grow as practitioners and researchers, and thus, our field will grow. I hope my reflections on the value of understanding lived experience in the context of behavioral feeding difficulties resonates. Accepting and embracing methods which enable us to inform our practice with lived experience will be key to working compassionately (Penney et al., 2023).

Writing this personal narrative has also been an insightful process and a venture into new qualitative methods. While I have always been open about my personal experiences, it *was* challenging finding what felt like an acceptable tone for a behavior analytic audience, in light of my prior experiences with qualitative research in the field. I wonder how an approach of this nature will be received, and face the same feelings of doubt—*is there any space in ABA for creative and non-traditional research methods?* This is the same question I faced when I embarked on my journey with qualitative research. My only conclusion is one in the spirit of Acceptance and Commitment Therapy—feel the fear and do it anyways. And I remain convinced, qualitative methods can grow our field in a new direction, if we let them.

## Data Availability

There is no data to make available.

## Footnotes

1 This quote was obtained as part of the author’s PhD research which is referenced throughout the paper. Ethical approval was obtained from the University of Kent for this study. Further information can be provided by contacting the author.

## Declarations

There were no human or animal participants in this paper.
